# Glucagonlike Peptide-1 Receptor Agonists and Asthma Risk in Adolescents With Obesity

**DOI:** 10.1001/jamanetworkopen.2025.51611

**Published:** 2025-12-29

**Authors:** Yung-Chieh Huang, Ming-Chin Tsai, Tim C. C. Lin, Lin-Shien Fu

**Affiliations:** 1 Division of Allergy, Immunology and Rheumatology, Department of Pediatrics, Taichung Veterans General Hospital, Taichung, Taiwan; 2 Department of Post-Baccalaureate Medicine, College of Medicine, National Chung Hsing University, Taichung, Taiwan; 3 Doctoral Program in Translational Medicine, National Chung Hsing University, Taichung, Taiwan; 4 Institute of Biomedical Science, National Chung-Hsing University, Taichung, Taiwan; 5 Department of Medical Research, China Medical University Hospital, Taichung, Taiwan; 6 Department of Medical Research, Taichung Veterans General Hospital, Taichung, Taiwan; 7 Department of Pharmacology, College of Medicine, Kaohsiung Medical University, Kaohsiung, Taiwan; 8 School of Medicine, National Yang Ming Chiao Tung University, Taipei, Taiwan

## Abstract

This cohort study investigates the association between glucagonlike peptide-1 receptor agonist use and the risk of acute asthma exacerbations among adolescents with overweight or obesity and asthma.

## Introduction

Overweight and obesity are established risk factors that contribute to greater severity and more frequent exacerbations of asthma among adolescents.^1^ Glucagonlike peptide-1 receptor agonists (GLP-1RAs) are increasingly prescribed for weight management in this population, but their effects on asthma control remain largely unknown. While some observational studies in adults have suggested a potential benefit,^2^ findings have been inconsistent,^3^ and no dedicated studies exist for adolescents. Therefore, this study aimed to investigate the association between GLP-1RA use and the risk of acute asthma exacerbations in a clinical cohort of adolescents with overweight or obesity.

## Methods

We conducted a retrospective cohort study using data from the TriNetX global federated health research network (January 1, 2020, to July 1, 2025). The study population included adolescents aged 12 to 18 years with concurrent diagnoses of asthma and overweight or obesity with or without diabetes (eFigure in Supplement 1). We compared patients with a new prescription for a GLP-1RA with a control group receiving documented nonpharmacological weight management interventions. Using 1:1 propensity score matching, we balanced the groups on baseline demographic characteristics, body mass index (BMI) categories, asthma severity proxies, and prior use of asthma- and diabetes-related medications (Table).^4^ The primary outcome was incidence of acute asthma exacerbation as defined by relevant *ICD-10* diagnosis codes. Secondary outcomes included systemic corticosteroid (SCS) prescriptions and emergency department (ED) visits for asthma. Further details are provided in the eMethods in Supplement 1. This study was approved by the Institutional Review Board of Taichung Veterans General Hospital and followed the STROBE reporting guideline. Given the anonymous nature of the deidentified data, the requirement for informed consent was waived.

**Table.** Baseline Cohort Characteristics Before and After PSM

Characteristic	Code^a^	Adolescents before PSM		Standardized difference	Adolescents after PSM		Standardized difference
		GLP-1RA (n = 547)	Control (n = 29 085)		GLP-1RA (n = 535)	Control (n = 535)	
Age at index, mean (SD), y	NA	15.8 (2.1)	14.3 (1.9)	0.783	15.8 (2.1)	15.8 (2.1)	0.03
Sex							
Female	NA	305 (55.8)	13 912 (47.8)	0.159	301 (56.3)	307 (57.4)	0.02
Male	NA	242 (44.2)	15 160 (52.1)	0.158	234 (43.7)	228 (42.6)	0.02
Unknown	NA	0	13 (0.04)	NA	0	0	NA
Race							
American Indian or Alaska Native	1002-5	10 (1.8)	229 (0.8)	0.092	10 (1.9)	10 (1.9)	<0.001
Asian	2028-9	10 (1.8)	929 (3.2)	0.087	10 (1.9)	11 (2.1)	0.01
Black or African American	2054-5	164 (30.0)	9275 (31.9)	0.158	159 (29.7)	139 (26.0)	0.08
Native Hawaiian or other Pacific Islander	2076-8	10 (1.8)	383 (1.3)	0.041	10 (1.9)	10 (1.9)	<0.001
White	2106-3	246 (45.0)	11 952 (41.1)	0.078	242 (45.2)	258 (48.2)	0.06
Unknown	NA	107 (19.6)	6299 (21.7)	NA	104 (19.4)	107 (20.0)	NA
Ethnicity							
Hispanic or Latino	2135-2	354 (64.7)	17 735 (61.0)	0.077	107 (20.0)	127 (23.7)	0.09
Non-Hispanic or non-Latino	2186-5	110 (20.1)	8266 (28.4)	0.195	347 (64.9)	331 (61.9)	0.06
Unknown	NA	83 (15.2)	3084 (10.6)	0.137	81 (15.1)	77 (14.4)	0.02
Socioeconomic status							
Adverse socioeconomic determinants of health	Z55-Z65	45 (8.2)	1555 (5.3)	0.115	45 (8.4)	46 (8.6)	0.01
Lifestyle-related problems	Z72	24 (4.4)	534 (1.8)	0.147	24 (4.5)	27 (5.0)	0.03
Family history of mental and behavioral disorders	Z81	10 (1.8)	321 (1.1)	0.06	10 (1.9)	10 (1.9)	<0.001
BMI distribution							
85th to 95th percentile	Z68.53	13 (2.4)	2604 (9.0)	0.287	13 (2.4)	16 (3.0)	0.04
95th to 120% of the 95th percentile	Z68.54	289 (52.8)	6970 (24.0)	0.622	279 (52.1)	311 (58.1)	0.12
120% to 140% of the 95th percentile	Z68.55	13 (2.4)	52 (0.2)	0.197	13 (2.4)	16 (3.0)	0.04
>140% of the 95th percentile	Z68.56	38 (6.9)	84 (0.3)	0.362	33 (6.2)	22 (4.1)	0.09
Unknown	NA	194 (35.5)	19 375 (66.6)	NA	197 (36.8)	170 (31.8)	NA
Preexisting asthma condition^b^							
Mild intermittent asthma	J45.2	175 (32.0)	8596 (29.6)	0.053	171 (32.0)	192 (35.9)	0.08
Mild persistent asthma	J45.3	86 (15.7)	3619 (12.4)	0.094	83 (15.5)	92 (17.2)	0.05
Moderate persistent asthma	J45.4	97 (17.7)	2816 (9.7)	0.236	93 (17.4)	102 (19.1)	0.04
Severe persistent asthma	J45.5	13 (2.4)	389 (1.3)	0.077	13 (2.4)	15 (2.8)	0.02
Other and unspecified asthma	J45.9	331 (60.5)	9573 (32.9)	0.576	322 (60.2)	244 (45.6)	0.30
Asthma exacerbation (in previous 1 y)							
Mild intermittent asthma with exacerbation	J45.21	24 (4.4)	1692 (5.8)	0.065	23 (4.3)	28 (5.2)	0.04
Mild persistent asthma with acute exacerbation	J45.31	10 (1.8)	635 (2.2)	0.025	10 (1.9)	12 (2.2)	0.03
Moderate persistent asthma with acute exacerbation	J45.41	16 (2.9)	786 (2.7)	0.013	16 (3.0)	17 (3.2)	0.01
Severe persistent asthma with acute exacerbation	J45.51	10 (1.8)	129 (0.4)	0.131	10 (1.9)	10 (1.9)	<0.001
Diabetes	E08-E13	115 (21.0)	747 (2.6)	0.597	109 (20.4)	111 (20.7)	0.01
Antidiabetic medication							
Metformin	6809	148 (27.1)	703 (2.4)	0.741	138 (25.8)	126 (23.6)	0.05
Insulin	HS501	47 (8.6)	433 (1.5)	0.329	44 (8.2)	54 (10.1)	0.07
SGLT2 inhibitors	A10BK	10 (1.8)	19 (0.1)	0.183	10 (1.9)	10 (1.9)	<0.001
Antiasthma medication							
Glucocorticoids, systemic	H02AB	211 (38.6)	7582 (26.1)	0.270	203 (37.9)	217 (40.6)	0.05
Glucocorticoids, inhaled	R03BA	246 (45.0)	10 479 (36.0)	0.183	240 (44.9)	240 (44.9)	<0.001
Selective beta-2 adrenoreceptor agonists, inhaled	R03AC	304 (55.6)	14 694 (50.5)	0.019	299 (55.9)	299 (55.9)	<0.001
Leukotriene receptor antagonists	R03DC	72 (13.2)	3118 (10.7)	0.068	70 (13.1)	62 (11.6)	0.05
Other systemic drugs for obstructive airway diseases	R03DX	10 (1.8)	90 (0.3)	0.148	10 (1.9)	10 (1.9)	<0.001
Antiobesity medication							
Phentermine	8152	19 (3.5)	71 (0.2)	0.241	16 (3.0)	16 (3.0)	<0.001
Topiramate	38404	50 (9.1)	455 (1.6)	0.341	46 (8.6)	38 (7.1)	0.06
Bupropion	42347	23 (4.2)	189 (0.6)	0.233	21 (3.9)	24 (4.5)	0.03
Naltrexone	7243	10 (1.8)	18 (0.1)	0.183	10 (1.9)	10 (1.9)	<0.001
Orlistat	37925	10 (1.8)	10 (0.03)	0.188	10 (1.9)	0	0.20
Total eosinophil count, µL							
0-200	LG32894-8	145 (26.5)	3450 (11.9)	0.379	139 (26.0)	158 (29.5)	0.08
200-400	LG32894-8	88 (16.1)	1903 (6.5)	0.305	82 (15.3)	88 (16.4)	0.03
>400	LG32894-8	50 (9.1)	1308 (4.5)	0.185	47 (8.8)	58 (10.8)	0.07
Unknown	NA	264 (48.3)	22 424 (77.1)	NA	267 (49.9)	231 (43.2)	NA

Abbreviations: PSM, propensity score matching; BMI, body mass index (calculated as weight in kilograms divided by height in meters squared); ED, emergency department; GLP-1RA, glucagonlike peptide-1 receptor agonist; NA, not applicable; SGLT-2, sodium-glucose cotransporter-2.

^a^ Codes are based on *International Statistical Classification of Diseases, Tenth Revision*, RxNorm, or SNOMED CT where applicable.

^b^ Categories are not mutually exclusive; patients may have diagnosis codes corresponding to more than 1 severity category recorded in the 12 months prior to the index date.

## Results

After propensity score matching, 1070 adolescents were included (mean [SD] age, 15.8 [2.1] years; 608 [56.8%] female and 462 [43.2%] male), with 535 each in the GLP-1RA group and control group, which were well-balanced by baseline characteristics (Table). During 12 months of follow-up, acute asthma exacerbations were significantly less frequent in the GLP-1RA group than in the control group (29 [5.4%] vs 57 [10.7%]; relative risk [RR], 0.51 [95% CI, 0.33-0.78]; *P* = .002). As shown in the Figure, use of GLP-1RAs was also associated with a lower incidence of asthma-related ED visits (8 [1.5%] vs 19 [3.6%]; RR, 0.42 [95% CI, 0.19-0.95]; *P* = .04) and SCS prescriptions (111 [20.7%] vs 168 [31.4%]; RR, 0.66 [95% CI, 0.54-0.81]; *P* < .001). Prescriptions for inhaled short-acting beta-2 agonists were also less frequent in the GLP-1RA group (173 [32.3%] vs 239 [44.7%]; RR, 0.72 [95% CI, 0.62-0.84]; *P* < .001) (Figure). Among patients who experienced at least 1 exacerbation, the mean (SD) number of subsequent events over 12 months was similar between the groups (1.83 [1.49] vs 2.02 [1.98]; *P* = .65).

**Figure.**
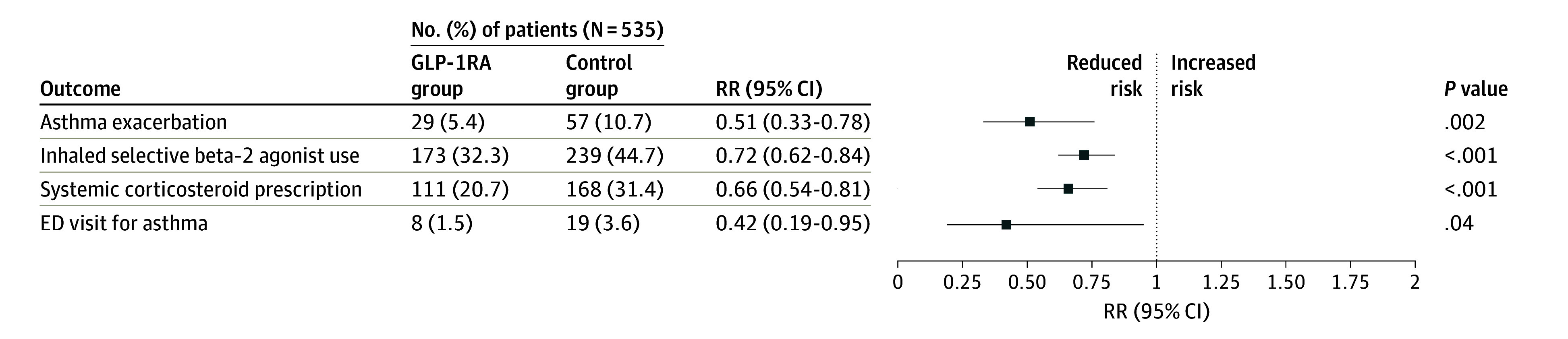
Association of Glucagonlike Peptide-1 Receptor Agonist (GLP-1RA) Use With Risk of Asthma Exacerbation and Other Asthma-Related Outcomes at 12 Months ED indicates emergency department; RR, relative risk.

## Discussion

To our knowledge, this study is the first to report an association between GLP-1RA use and a lower risk of acute asthma exacerbations in adolescents with overweight or obesity. Our findings suggest a potential dual benefit for this population, where a single class of medication could address both weight management and lower risk for asthma exacerbation, thereby potentially reducing the burden of 2 common and interconnected chronic conditions.

An important question is whether the observed association reflects weight loss or weight-independent anti-inflammatory effects of GLP-1RAs.^5,6^ Although metabolic dysfunction and insulin resistance are common in obesity-related asthma, our data include BMI categories rather than individual longitudinal BMI changes. Thus, we cannot determine the underlying mechanism, and prospective studies are needed to clarify whether these respiratory benefits are independent of weight loss.

The primary limitations of this study are its retrospective, observational design, which precludes causal inference, the potential for residual confounding from unmeasured variables and factors not fully captured by the proxies used for matching, and its reliance on a predominantly US-based database, which may limit external validity to other countries. These hypothesis-generating findings warrant confirmation in prospective randomized clinical trials to establish the efficacy and safety of GLP-1RAs as a potential adjunct therapy for asthma in adolescents with overweight or obesity.

## Notes

Supplement 1. **eMethods.** Study Population and Analysis **eFigure.** Flowchart of Patient Selection

Supplement 2.
Data Sharing Statement
