# Supplementary material for: Glucagonlike Peptide-1 Receptor Agonists and Asthma Risk in Adolescents With Obesity
Source: JAMA Netw Open. 2025 Dec 29;8(12):e2551611. doi: 10.1001/jamanetworkopen.2025.51611 (PMC12750248; doi:10.1001/jamanetworkopen.2025.51611)
Supplement: Supplement 1. — eMethods. Study Population and Analysis eFigure. Flowchart of Patient Selection [file jamanetwopen-e2551611-s001.pdf]

Supplementary Online Content

Huang YC, Tsai MC, Lin TCC, Fu LS. Glucagonlike peptide-1 receptor agonists and asthma risk in adolescents with obesity. *JAMA Netw Open.* 2025;8(12):e2551611. doi:10.1001/jamanetworkopen.2025.51611

eMethods. Study Population and Analysis
eFigure. Flowchart of Patient Selection

This supplementary material has been provided by the authors to give readers additional information about their work.

© 2025 Huang Y-C et al. *JAMA Network Open*.

eMethods. Study Population and Analysis

Database

This is a retrospective cohort study using data acquired from TriNetX, a global federated health research network providing access to de-identified electronic medical records from large healthcare organizations (HCOs). As of July 2025, the network comprises 156 HCOs. For this study, data were contributed by 120 HCOs that provided eligible records; approximately 99% of these contributing HCOs are located in the United States**.** The study was approved by the Institutional Review Board of Taichung Veterans General Hospital (IRB no. CE23454A), and the requirement for informed consent was waived.

Cohort Definition and Index Date:

The study population included adolescents aged 12 to 18 years between January 1, 2020, and July 1, 2025.

Cohort Entry and Asthma Definition: Patients were required to have both a diagnosis of asthma (ICD-10 code: J45) and a diagnosis of overweight or obesity (ICD-10: E66 or Z68.53-56) present in their record during the study window. We acknowledge that this definition, based on the presence of a code, cannot reliably distinguish the date of initial asthma diagnosis from the coding of a pre-existing condition.

To mitigate potential confounding from asthma history and varying disease activity, our matching model (described below) explicitly controlled for crucial proxies of baseline asthma severity. These included a defined preexisting asthma condition (using the ICD-10 sub-classifications; J45.2 mild intermittent, J45.3 mild persistent, J45.4 moderate persistent and J45.5 severe persistent) and baseline use of anti-asthma medications (including systemic/inhaled glucocorticoids and rescue inhalers) in the year prior to the index date.

Index Date and Follow-up: We employed a "new-user" design for the exposure group and a "new-intervention" design for the control group to ensure parallel timing and mitigate time-related biases. [4]

- For the GLP-1RA group, the index date was the date of the first prescription for liraglutide (RxNorm: 475968) or semaglutide (RxNorm: 1991302).
- For the Control group, the index date was the date of the first documented non-pharmacological weight management intervention, including dietary (ICD-10 Z71.3, CPT 1013548, SNOMED 61310001, 386373004, 266724001), exercise (ICD-10 Z71.82), or behavioral (HCPCS G0447) codes.

© 2025 Huang Y-C et al. *JAMA Network Open*.

Follow-up for all outcomes began on the index date and continued for 12 months. This methodological approach is consistent with other high-impact, real-world studies using this platform. Patients in both groups were required to have at least one follow-up visit within 3 months after the index date to ensure active participation in the health system. Patients with any documented use of GLP-1RA before age 19 were excluded from the control group.
The patient selection process is illustrated in eFigure 1.

Covariates and Outcomes
Baseline Covariates: We collected baseline characteristics in the 12 months prior to the index date, including age, sex, race/ethnicity, BMI category (overweight = ≥85th to <95th percentile; obesity class 1 = ≥95% to <120% of the 95th; class 2 = ≥120% to <140% of the 95th; class 3 = ≥140% of the 95th), preexisting asthma condition, a diagnosis of diabetes mellitus, and baseline eosinophil count. We also captured baseline use of anti-diabetic medications (metformin, insulin, SGLT-2 inhibitors), anti-obesity medications (phentermine, topiramate, bupropion, naltrexone, orlistat) and anti-asthma medications (systemic glucocorticoids, inhaled glucocorticoids, inhaled selective beta-2-adrenoreceptor agonists, leukotriene receptor antagonists).
Outcomes: The primary outcome was the incidence of acute asthma exacerbation within 12 months since the index date. This was defined by relevant ICD-10 diagnosis codes for acute asthma exacerbation (ICD10 J45.21, J45.31, J45.41, J45.51, J45.901). This was analyzed as a binary, patient-level outcome; patients were considered to have met the outcome if they had one or more (≥1) qualifying exacerbation codes during the 12-month follow-up period.
Secondary outcomes reported in the main text included: (1) systemic corticosteroid prescriptions, (2) asthma-related emergency department (ED) visits, and (3) prescriptions for inhaled short-acting β2-agonists.
Statistical Analysis
We performed 1:1 propensity score matching (PSM) using the TriNetX platform's built-in algorithm.
The PSM model included the following baseline covariates:

- Age at index event
- Sex, race, and ethnicity
- Socioeconomic determinants of health (as categories)
- Preexisting asthma condition (by sub-classification: mild intermittent, mild persistent, moderate persistent, severe persistent) and asthma exacerbation (in previous one year) (by sub-classification)

© 2025 Huang Y-C et al. *JAMA Network Open*.

- BMI category (overweight = ≥85th to <95th percentile; obesity class 1 = ≥95% to <120% of the 95th; class 2 = ≥120% to <140% of the 95th; class 3 = ≥140% of the 95th)
- Diagnosis of diabetes mellitus
- Baseline eosinophil count (as categories)
- Baseline use (yes/no) of all anti-diabetic, anti-obesity and anti-asthma medications listed in the Baseline Covariates section above.

Categorical variables were compared using chi-square tests, and continuous variables using t-tests. Risk ratios (RRs) with 95% CIs were generated. For patients who experienced at least one exacerbation, the mean number of events was calculated and compared using a two-sample t-test. Missing data for categorical covariates (e.g., eosinophil count) were treated as a distinct category in the matching model.

eFigure. Flowchart of Patient Selection.

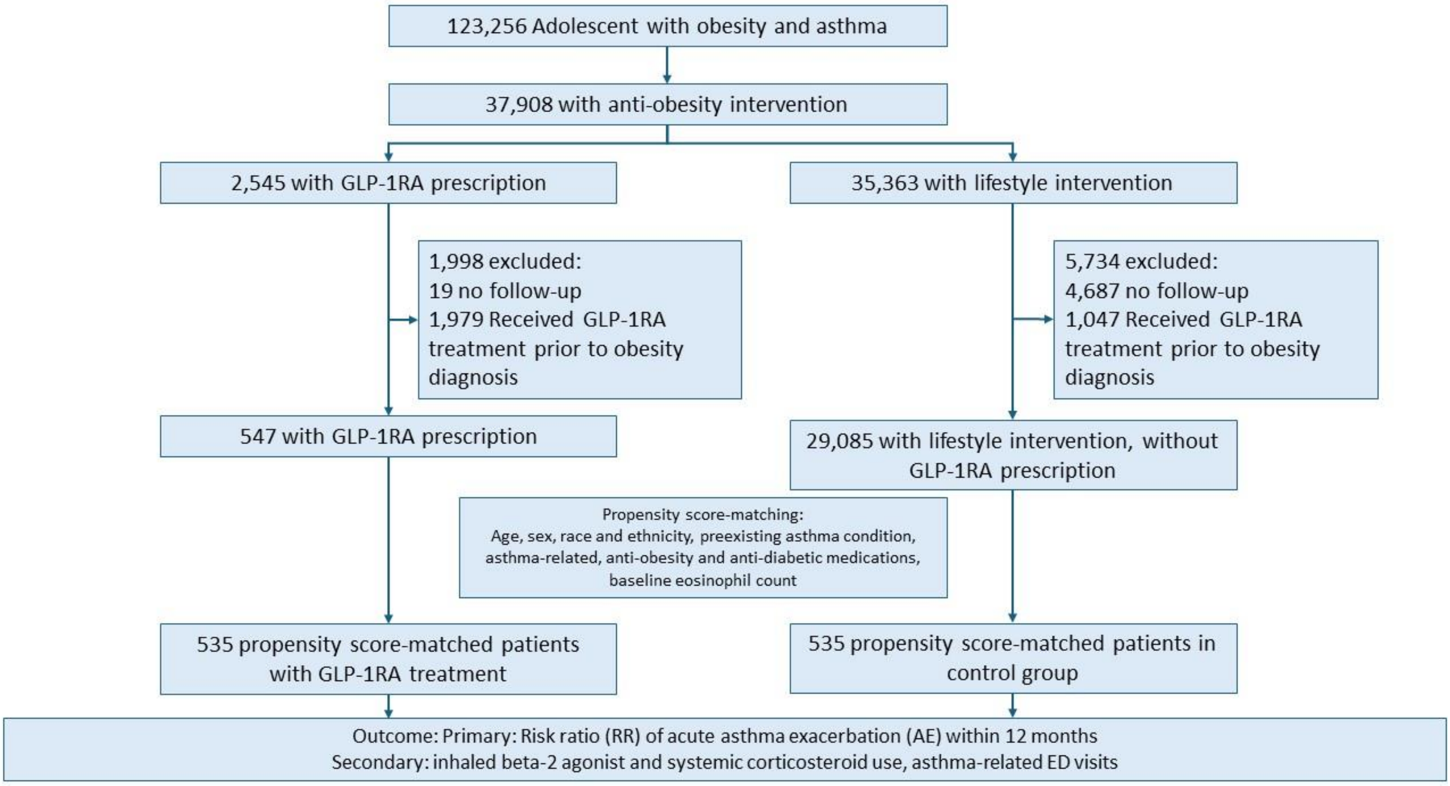

© 2025 Huang Y-C et al. *JAMA Network Open*.
