# Supplementary material for: Glucagonlike Peptide-1 Receptor Agonists and Asthma Risk in Adolescents With Obesity
Source: JAMA Netw Open. 2025 Dec 29;8(12):e2551611. doi: 10.1001/jamanetworkopen.2025.51611 (PMC12750248; doi:10.1001/jamanetworkopen.2025.51611)
Supplement: Supplement 2. — Data Sharing Statement [file jamanetwopen-e2551611-s002.pdf]

Data Sharing Statement

Huang. Glucagonlike Peptide-1 Receptor Agonists and Asthma Risk in Adolescents With Obesity. *JAMA Netw Open*. Published December 29, 2025.
doi:10.1001/jamanetworkopen.2025.51611

Data
Data available: No

Additional Information
Explanation for why data not available: The conditions under which the data were provided do not allow for the data to be made publicly available. The data we used for this paper were acquired from TriNetX (https://www.trinetx.com/). Release and/or sharing of these data are not covered under our data use agreement with TriNetX. All the required information to replicate the network queries is presented in the manuscript. To gain access to the network, a request can be made to TriNetX (join@trinetx.com), but costs may be incurred, and a data sharing agreement would be necessary.
